# The impact of antiviral therapy on hepatocellular carcinoma epidemiology

**DOI:** 10.2217/hep-2017-0024

**Published:** 2018-05-10

**Authors:** Massimo Colombo, Ana Lleo

**Affiliations:** 1 Department of Internal Medicine, Center for Translational Research in Hepatology, Humanitas Clinical and Research Center, 20089 Rozzano (MI), Italy; 2 Department of Biomedical Sciences, Humanitas University, Via R. Levi Montalcini 4, 20090 Pieve Emanuele (MI), Italy; 3 Hepatology, Department of Internal Medicine, Humanitas Clinical & Research Center, Rozzano (MI), Italy

**Keywords:** direct-acting antivirals, HBV, HCV, hepatocellular carcinoma, nucleos(t)ide analogs

## Abstract

The development of nucleos(t)ide analogs and direct antiviral agents has revolutionized the management of chronic infection with HBV and HCV, respectively. These regimens allow to expand treatment to virtually all infected, including those with poor hepatic reserve and those with severe comorbidities. As a result, permanent suppression of HBV and eradication of HCV has been achieved in almost all treated patients, resulting in substantial clinical benefits. In several cohorts, these successes have translated into a reduction of the incidence of hepatocellular carcinoma that was more frequently observed in patients with less advanced hepatitis, whereas liver cancer was more often associated with male gender, cirrhosis, alcohol abuse and diabetes.

**Practice points**

- Hepatocellular carcinoma (HCC) must be diagnosed by pathology or by noninvasive criteria according to international guidelines.
- Hepatitis B virus (HBV) patients under effective long-term nucleos(t)ide analog therapy should remain under surveillance for HCC. HCC surveillance is mandatory for all patients with HBV-related cirrhosis as well as those with moderate or high HCC risk scores at the onset of nucleos(t)ide analog therapy.
- Sustained virological response after hepatitis C virus (HCV) antiviral treatment, results in improved clinical outcomes, including lower risk decompensation and HCC development; HCC surveillance is mandatory for all patients with HCV-related cirrhosis even after HCV clearance.
- Hepatic decompensation is the major driver of death in HCV cirrhotic patients with successfully treated early HCC, HCV patients should have access to early direct acting antiviral treatment.

Hepatocellular carcinoma (HCC) stands as a most difficult to cure end-stage liver disease and the dominant cause of death of patients with compensated cirrhosis of any etiology [1]. In 2012, the death toll of HCC was calculated in 521,000 men and 224,500 women who have died from liver cancer. Although HCC is the second leading cause of cancer death in men and the sixth in women [2], a recent WHO report indicates that this cancer is globally on the rise as a consequence of a restless growth of the world population. Indeed, from 1990 to 2013, the global burden of HCC has increased by 60%, and the relative incremental fraction attributed to hepatitis B and hepatitis C was 51 and 290%, respectively [3]. In both hemispheres, 60 to 80% of all patients with HCC present with an underlying liver disease caused by either HCV or HBV [4]. Not surprisingly, therefore, the prevalence of HCC has long been lower in developed regions than in developing regions, where viral hepatitis prevails, yet an analysis of the WHO database in 2012 disclosed HCC mortality to be on the rise in northern Europe and North America, mainly as a consequence of epidemics of viral hepatitis spread through parenteral risk behaviors [5,6]. Conversely, in traditionally high risk countries including Mediterranean European countries, Japan, and Hong Kong, the attack rates of liver cancer caused by viral hepatitis have been attenuated at population level through effective public health interventions (i.e., massive HBV vaccination for the young generation, HBV education and screening and the highly effective nucleos(t)ide analogs) [7] whereas projections have been made for a decline of HCC mortality following massive access of infected patients to antiviral therapy [8]. Last year, the WHO launched a program for the elimination of viral hepatitis by 2030 with the aim of reducing by 65% hepatitis-related mortality, which in part is caused by HCC.

## Impact of HBV antiviral therapy on hepatocellular carcinoma epidemiology

HBV is known to be a strongly oncogenic virus, as it triggers changes in gene expression that confer the hallmarks of cancer on hepatocytes. The pathogenesis of HBV-related HCC is a multifactorial process, which involves an increased genomic instability due to HBV DNA integration into host genome, direct effect of HBV proteins and a critical immune component [9,10]. The environment created in the liver by immune responses is exploited by HBV to activate signaling pathways that block apoptosis, support HBV replication and facilitates virus persistence. HBV may also delay or block innate immunity, thereby influencing the adaptive immune responses. The direct HBV oncogenic activity is further enhanced by chronic active inflammation through increased oxidative stress, necrosis, regeneration, angiogenesis and cellular senescence [11].

Although the natural history studies in untreated HBV patients have reported a 5-year cumulative risk of developing hepatocellular carcinoma of 17% in east Asia and 10% in the western Europe and the USA [12], development of effective antiviral therapies has dramatically changed the outcome of HBV patients [13]. Given that the ultimate goal of the treatment of chronic hepatitis B is the prevention of advanced liver disease, including both cirrhosis and HCC, the majority of chronic hepatitis B patients are currently treated with an oral antiviral agent. The current first-line nucleos(t)ide analogs (NAs), entecavir and tenofovir disoproxil fumarate, are highly effective in suppressing HBV replication and have a high barrier to resistance with minimal or no risk of viral resistance in the long-term and are therefore recommended for the optimal management of HBV infection [14].

The natural history of chronic HBV infection rests on three major milestones, beginning with HBeAg clearance, followed by the seroclearance of HBV DNA, and eventually ending with HBsAg clearance; under antiviral treatment, the usual course tends to be first clearance of HBV DNA, followed by HBeAg loss or conversion to anti-HBe and eventually HBsAg loss or rarely HBsAg seroconversion. Importantly, while NA therapy inhibits HBV replication, it does not eradicate HBV from the liver and HBsAg is still detectable in serum of almost all patients under NA therapy, a fact that makes reasonable to expect a reduction but not an elimination of the HCC risk. Large studies in Asia, including matched untreated controls, have shown that NA treatment, and particularly treatment-induced virologic suppression, significantly reduces the risk of HBV-related HCC [15–18]. Despite the important risk reduction determined by NA therapy, annual HCC incidences range from 0.9 to 5.4% in patients who have already developed cirrhosis and have been treated with first-line recommended NAs [13].

Although clearance of serum HBV DNA significantly associates to a reduced risk of developing HCC, serum HBsAg levels have been able to predict HCC risk among those with undetectable viral loads, with the lowest HCC risk among those with low HBsAg levels (<1000 IU/ml) [19]. Importantly, prevention of HBV-related HCC by NA therapy is strongly associated with treatment duration and older age. A recently published multicentric European cohort study including 1951 adult Caucasian patients without HCC at baseline, demonstrated that the HCC risk decreases beyond year 5 of NA therapy, particularly in those with compensated cirrhosis [20]. The authors show a yearly HCC incidence rate of 1.22% within and 0.73% after the first 5 years, the yearly HCC incidence rate did not differ within and after the first 5 years in noncirrhotic, but it significantly declined in cirrhotic subjects (3.22 vs 1.57%). Importantly, all HCCs beyond year 5 developed in patients older than 50 years at NA onset. Nonetheless, as the duration of antiviral treatment increases, there is a residual risk for HCC in spite of undetectable HBV DNA in serum for 12.4 years, as recently reported [21]. Corroborating the findings in cohort studies were real-life investigations like the national viral hepatitis therapy program that was launched in Taiwan in October 2003 where, by 2011, a total of 157,570 patients had received therapy for chronic hepatitis B. In that study, Chiang and colleagues demonstrated that a strong decline of HCC following NA therapy as the lifetime (30–78 years old) risk of developing HCC was as high as 21.7%. Interestingly, mortality and incidence rates of HCC decreased continuously from 2000 to 2003 (before therapy program) through 2004–2007 to 2008–2011 in all age and gender groups [22].

## Chronic HCV infection & hepatocellular carcinoma prevention

The patients with HCV-related cirrhosis have an expected high rate of progression to liver decompensation, HCC, and eventually death [23]. Numerous studies, mostly linked to interferon-treated patients, have demonstrated that the achievement of sustained virological response (SVR) after antiviral treatment results in improved clinical outcomes, including lower risk decompensation and HCC development [24,25]. Van der Meer and colleagues have recently published a large retrospective observational study including 1000 pooled cirrhotic patients with SVR after interferon-based therapy; after a median follow-up of 5.7 years (interquartile range: 2.9–8.0), patients with HCV-induced cirrhosis and SVR showed an annual risk of approximately 1% for HCC [26].

Further, compensated cirrhotic patients with SVR to an interferon-based regimen have been demonstrated to have a survival rate similar to that of the age-and gender-matched general population [27] and lower risk of HCC occurrence [27,28]. These studies include large cohorts with long-term follow-up that lead to solid clinical information; however, interferon-based therapy is no longer a standard of care and included patients were affected of well compensated cirrhosis with moderate portal hypertension.

Direct acting antiviral (DAA) agents have revolutionized the treatment of HCV infection, with very high rates (>90%) of SVR and good safety profile also in the advanced stages of disease. Currently, DAAs are the accepted standard of cure in HCV patients, including those with advance liver disease and previous HCC. However, data on the benefit of viral eradication after DAAs on advance disease, disease progression and liver-related complications, including HCC, are still limited and controversial. Recently, the enthusiasm for NAs to treat HCV has been clouded by two retrospective observational reports suggesting that treatment of HCV with DAAs could increase the risk HCC early recurrence in patients with early stage of HCC. The BCLC group reported a surprisingly high rate (28%) of earlier than expected HCC recurrences in the patients with a small HCC cured by radical therapies (i.e., resection or local ablation) [29]; a finding that was confirmed by a group in Italy who published an early HCC recurrence in 17 out of 59 (29%) patients after DAA treatment [30]. Mitigating the concern raised by these reports, however, was the perception of potential biases of the study design of these retrospective reports; including baseline patients and tumor characteristics, type of curative HCC treatment, assessment of complete radiological response, and time frames between tumor cure and DAA therapy. Furthermore, the parallel publication of more than one study from different geographical regions, including a large multicenter ARNS study in France and the large VA-cohort, provided opposite interpretation regarding the risk of HCC occurrence and recurrence after DAA therapy [31,32]. In more than one study, a relationship was demonstrated between HCC occurrence following DAA therapy, duration of the post treatment surveillance period, patient age, and severity of the liver impairment. A large systematic review, meta-analyses, and meta regression, comparing the rate of HCC occurrence in patients with HCV-related cirrhosis following DAA versus interferon-based cure was recently published [33]. The authors aimed to compare the rate of HCC recurrence in the patients who received curative HCC treatment, following DAA versus interferon-based cure and included a total of 13,875 patients from 41 studies. Despite the limits of the methodology itself, the authors concluded that there is no evidence for differential HCC occurrence or recurrence risk following SVR from DAA and interferon-based therapy.

Importantly, it was recently demonstrated that hepatic decompensation is the major driver of death in HCV cirrhotic patients with successfully treated early HCC [34]; the authors therefore suggest that HCV eradication after treatment with new DAA agents could improve overall survival through long-term preservation of liver function.

## Will antiviral therapy of HBV & HCV impact on HCC epidemiology?

Whether the undisputed clinical benefits of antiviral therapy have translated into a reduction of HCC burden is debated. This in part relates to the fact that registry coverage of the world population is very limited, but also because the number of patients with HBV or HCV (Table 1) achieving a cure is currently counterbalanced, if not outnumbered, by a growing epidemic of new infections, mainly related to risky behaviors [8]. In 2003, a national program for treatment of patients with chronic hepatitis B was launched in Taiwan, a hyperendemic area for HBV which in 1984 successfully pioneered a campaign of mass vaccination of newborns against HBV [22]. Owing to the existence of a nationwide registry that captures all diagnoses as well as delivery and outcomes of therapies, the effectiveness of the vaccination campaign was readily measurable in terms of a decline of HBV carriage state among the youngest generation; importantly, the implementation of the national program for treatment of viral hepatitis required adjustments of reimbursement policies for the treatment of HBV. By 2011, more than 150,000 Taiwanese had received effective NA therapy resulting in a measurable decline of HCC incidence as well that was accompanied by a substantial decline of mortality for chronic liver disease and liver cancer [22]. Compared to the period 2000–2003, between 2008 and 2011 the age and gender adjusted relative ratio of HCC mortality was 0.76 and the relative ratio of HCC incidence was 0.86. Similar figures are not yet available for HCV, owing to the late arrival on the market of user friendly interferon-free regimens that have eased patient access to anti-HCV therapy and the existence of budgetary constraints that in many regions prevented scaling up of strategies for treatment of HCV infected individuals. Although the latter constraints will soon be bypassed by widespread patient access to low-priced generics, there are a number of barriers still in place that jeopardise the success of the WHO programme for elimination of HBV and HCV by 2030.The most important one is the lack of an accurate estimation of the global burden of viral hepatitis due to the lack of screening programs for viral hepatitis in the general population worldwide. The rates of diagnosis, in fact, are globally strikingly low, totaling 9% of 240 million people who are chronically infected with HBV and 20% of 70 million people with chronic hepatitis C [35]. A second constraint are the low rates of cures weighed against the annual rates of new infections with HBV and HCV. In the HBV scenario, only 10% of the infected population is under treatment or has received interferon-based therapies, and strategies of treatment as prevention of transmission that are considered of strategic importance in the fight against viral hepatitis are almost fully implemented in the setting of mother to child transmission only, but unfortunately are not in the area of harm reduction policies. In the HCV arena, 10% of the infected population has successfully been treated with antivirals worldwide, including those who in the past received interferon-based regimens. Scale up of anti-HCV therapy, however, needs to be globally implemented as an epidemiological study in 91 representative countries indicated that HCV elimination by 2030 would require that each year the cumulative number of cured patients and deaths due to end stage HCV exceeds by 7% the number of newly infected people, which totals 1.7 million cases per year [36]. Currently, as very few countries are on target to achieve elimination of HCV as a public health problem by 2030, a parallel decline of HCC incidence, as a consequence of antiviral therapy, is expected to take place at a quite low pace.

**Table 1.** **The HCV epidemiology calculated in 2016 versus 2017.**

**Region**	**HCV epidemic 2016**	**New HCV infections**	**Number cured**	**HCV-related deaths**	**HCV epidemic 2017**	**Net change**
Asia and Pacific	29,564,900	574,330	456,552	179,810	29,502,868	-62,032

Central and eastern Europe	6,507,700	322,800	26,110	15,505	6,788,885	+281,185

Latin and South America	3,477,400	27,537	47,859	21,496	3,435,582	-40,548

North Africa and Middle East	7,399,470	156,660	542,724	51,944	6,961,462	-438,008

North America	2,955,600	31,870	216,731	20,829	2,749,910	-205,690

Sub-Saharan Africa	5,069,000	130,800	3805	21,540	5,174,455	+105,455

Western Europe	2,364,430	35,440	105,821	14,951	2,279,098	-85,332

Subtotal of 91 countries	57,338,500	1,279,437	1,399,602	326,075	56,892,260	-446,240

Missing countries	12,216,308	318,375	113,157	57,923	12,363,603	+147,295

Global estimate	69,554,808	1,597,812	1,512,759	383,998	69,255,863	298,945

HCV: Hepatitis C virus

Reproduced with kind permission from [36].

## Conclusion

Implementation of sanitation policies is the pillar of prevention of viral hepatitis spread among the general population. Permanent suppression of HBV and eradication of HCV have been achieved in almost all treated patients, resulting in substantial clinical benefits, prevention of advanced liver disease due to HBV and HCV, and reduction of the global burden of HCC. There are still a number of barriers in place that jeopardize the success of the WHO program for elimination of HBV and HCV by 2030. The most important one is the lack of screening programs for viral hepatitis in the general population worldwide. Clearance of serum HBV DNA significantly associates to a reduced risk of developing HCC, however, there is a residual risk for HCC in spite of undetectable HBV DNA in serum.

Direct-acting antiviral agents have revolutionized the treatment of HCV infection, with very high rates (>90%) of sustained virological response and also a good safety profile in the advanced stages of disease. Currently, direct-acting antivirals are the accepted standard of cure in HCV patients, including those with advanced liver disease and previous HCC.

Widespread access to generics remains the only pragmatic approach to scale up antiviral therapy of infected individuals in resource-poor regions.

## Future perspective

Implementation of sanitation policies, like screening of blood donors and harm reduction strategies, is the pillar of prevention of viral hepatitis spread among the general population. This, coupled with a scale up in antiviral therapy of the infected individuals, will enhance prevention of advanced liver disease due to viral hepatitis B and C, and consequently lead to a reduction of the global burden of HCC. However, the massive access to generics remains the only pragmatic approach to scale up antiviral therapy of infected individuals in the resource poor regions.
